# Supplementary material for: Improvement in the Sustained-Release Performance of Electrospun Zein Nanofibers via Crosslinking Using Glutaraldehyde Vapors
Source: Foods. 2024 May 20;13(10):1583. doi: 10.3390/foods13101583 (PMC11121536; doi:10.3390/foods13101583)
Supplement: Supplementary file 1 [file foods-13-01583-s001.zip › foods-2983898-supplementary.pdf]

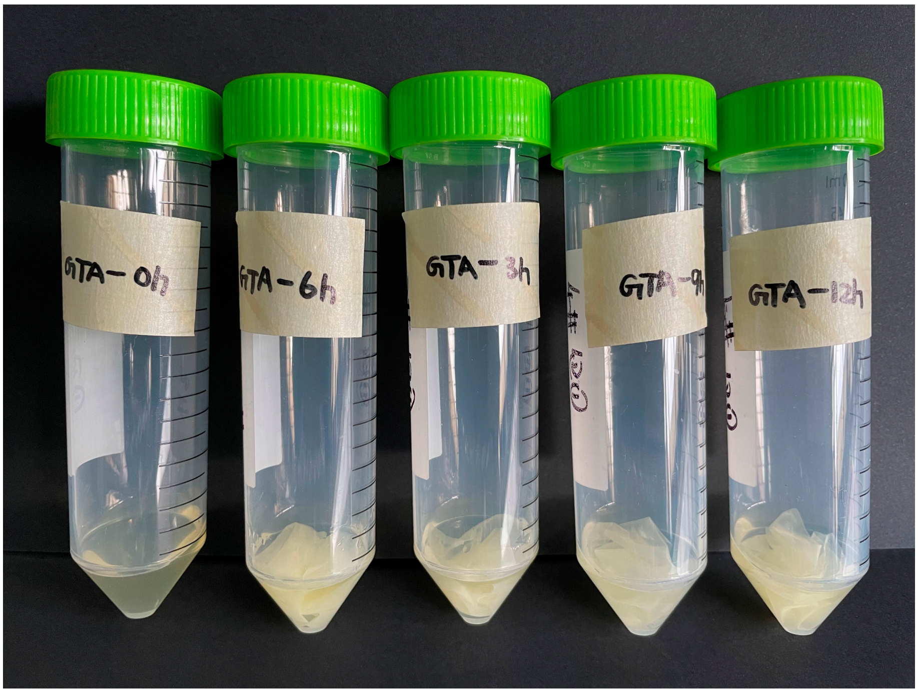

Figure S1. Macroscopic images of as-spun and crosslinked zein nanofibers exposed to acetic acid for 24 h.

Table S1. Average fiber diameters and standard deviation of non-crosslinked and GTA crosslinked zein nanofibers.

Samples	Average fiber diameters (nm)	standard deviation
GTA_0h	208.22	46.93
GTA_3h	225.52	47.25
GTA_6h	224.32	52.93
GTA_9h	226.96	68.00
GTA_12h	267.69	90.97

Table S2. Functional groups and structural changes in the as-spun and crosslinked zein nanofibers [41].

Characteristic peaks	Functional groups	Samples				
		GTA_0h	GTA_3h	GTA_6h	GTA_9h	GTA_12h
1	N-H and O-H stretching vibration (Amide A)	3295 cm^{-1}	3292 cm^{-1}	3292 cm^{-1}	3276 cm^{-1}	3275 cm^{-1}
3	C=O stretching vibration (Amide I)	1653 cm^{-1}	1653 cm^{-1}	1652 cm^{-1}	1648 cm^{-1}	1648 cm^{-1}
4	N-H bending vibration (Amide II)	1542 cm^{-1}	1542 cm^{-1}	1542 cm^{-1}	1542 cm^{-1}	1542 cm^{-1}
5	symmetric stretching vibration of the C=O groups	-	1077 cm^{-1}	1066 cm^{-1}	1066 cm^{-1}	1066 cm^{-1}
